# Editorial: Bioactive compounds from medicinal mushrooms and plants - extraction and potential application in foods

**DOI:** 10.3389/fnut.2025.1762037

**Published:** 2026-01-09

**Authors:** Aleksandra Sknepnek, Dunja Miletić, Aleksandra Cvetanović Kljakić

**Affiliations:** 1 Faculty of Agriculture, Institute of Food Technology and Biochemistry, University of Belgrade, Belgrade, Serbia; 2 Faculty of Technology Novi Sad, University of Novi Sad, Novi Sad, Serbia

**Keywords:** bioactive compounds, by-products, mushrooms, natural products, plants

Medicinal mushrooms and medicinal plants have been used for centuries as valuable sources of various bioactive compounds. Consequently, they are widely recognized as alternative therapies for health promotion and the treatment of various diseases (1). Additionally, the antioxidant and antimicrobial effects of bioactive compounds may allow their use in foods to replace synthetic additives, thereby improving safety, food quality, and shelf life. Although the literature on plants and mushrooms contains numerous studies on their chemical composition and potential biological activities, many plants, mushrooms, and their by-products remain insufficiently explored, despite their possible nutritional or medicinal value.

Extensive agro-industrial exploitation of natural resources leads to significant accumulation of waste and its release into the ecosystem. Its recycling approach can significantly contribute to global economic development, besides protecting the environment. Several articles on this Research Topic have addressed by-products from the agro-forestry sector as potential sources of bioactive components with various applications. Avocado peel (AVDP) was the focus of Oke et al.'s review, highlighting it as a low-cost source of phenolic compounds with applications in the functional food industry, pharmaceuticals, and cosmeceuticals. Due to the increasing demand for alternatives to synthetic substances, AVDP contributes to food quality and shelf life, as a substitute for synthetic additives. Zirari et al. aimed to determine the potential nutritional, antioxidant, and medicinal value of the woody parts of the plant *Abies marocana*. Their research showed that these woody parts have significant nutritional potential, and their extracts exhibit strong antioxidant potential, with no acute toxicity. In another study, Wang et al. identified nine phenolic compounds in cedar pine needles for the first time by fractionating extracts according to polarity, using a methodology that enabled linking these compounds to anti-obesity effects and demonstrated that phenolic acids are the primary bioactive molecules. The significance of this research lies in its effective approach for separating compounds of similar polarity and its focus on small, widely distributed plant metabolites that are often overlooked due to their simple structure.

The articles published in this Research Topic also address techniques used in post-harvest treatments, as these can affect the structure, biological activities, nutritional, and medicinal value of bioactives. To achieve optimal extraction efficiency of the target compound, the extraction technique must be selected based on the specific mushroom or plant type and the physicochemical properties of the compound. In addition, unprotected natural compounds can lose quality and stability under adverse environmental conditions. Therefore, different encapsulation techniques can be used to increase stability, bioavailability, and controlled release while reducing toxicity (2). Xiao et al. investigated the influence of three drying techniques on the proteome profiles of *Cordyceps sinensis* mushroom using the 4D-DIA method. Minimal variance in proteins was noted in dried compared to fresh sample when the freeze-drying method was applied, while the use of higher temperatures (oven and air drying) deteriorated proteins, altering their structure, which can consequently lead to changed and reduced medicinal properties of the mushroom. In a comprehensive review, Gorrepati et al. presented *Allium* species as an insufficiently examined multifunctional group of herbs with broad pharmacological applications. They provided an up-to-date overview of specific bioactive compounds originating from different plant organs and their medicinal properties. They also offered insight into new extraction techniques and stabilization methods for *Allium* species to improve outcomes. In the research by Taşkın et al., 70% crude ethanolic extracts of *Sideritis germanicopolitana* subsp. *viridis* (SGV) and *Sideritis libanotica* subsp. *linearis* (SLL) demonstrated better antioxidant, antiurease and anticholinesterase properties than chloroform and petroleum ether extracts. Although, the crude plant extracts also showed better activity than extracts loaded in nanoparticles, *in vitro* release studies proved that SGV and SLL nanoparticles are capable of drug delivery applications. Due to the many advantages of carrier system applications, the encapsulated formulations of SGV and SLL extracts increased their potential for use in real-life treatments.

A diverse array of primary and secondary metabolites from plants and mushrooms exhibit various biological activities. Considering that around 50% of pharmaceutical drugs originate from natural products, these are recognized as key contributors to drug discovery (3). Bioactive compounds of natural origin have a long-term safety record compared to their synthetic pharmaceutical and/or nutraceutical alternatives. In a review by Tang et al., the authors showed that kaempferol—a flavonoid phenolic compound—is at the forefront of natural product research. Since 2022, over 1,000 articles have been published annually on this topic. The review identified the leading contributing countries and the most studied biological mechanisms, while also highlighting emerging application areas such as cardiovascular protection, neuroprotection, and antiviral and antibacterial effects. In a systematic review, Xu et al. provided a theoretical basis for the development of sea buckthorn (*Hippophae Fructus*) products in the field of anti-tumor and clinical applications. Sea buckthorn is a food and medicinal species whose fruits, leaves, and by-products contain active compounds such as isorhamentin, quercetin, gallic acid, and protocatechuic acid, which exhibit antitumor effects and liver protection. In a review, Sknepnek et al. highlighted the potential of bioactive compounds from plants and mushrooms, including polyphenols, terpenes, terpenoids, alkaloids, polysaccharides, proteins, and peptides, their mechanisms of action, and the importance of extraction techniques to obtain the highest biological and therapeutic potential in the prevention, management, and treatment of type 2 diabetes mellitus. In another systematic review, the Zhang et al. focused on plant-based foods rich in phytosterols—a natural triterpene compound—in combating hyperlipidemia. This chronic metabolic disorder represents a significant risk factor for atherosclerotic cardiovascular diseases. The analysis, which included 1,088 participants from 14 randomized controlled trials, revealed that sterol-rich foods can reduce low-density lipoprotein cholesterol (LDL-C) and total cholesterol (TC), while increasing high-density lipoprotein cholesterol (HDL-C) levels in patients with hyperlipidemia. In a research article by Lin et al., the authors addressed ulcerative colitis (UC), a disease caused by chronic inflammation and ulceration of the inner mucosal lining of the rectum and colon. Multiple factors contribute to the onset and development of the disease, including environmental, genetic, lifestyle, and gut-related factors (4). A study published in this Research Topic demonstrated the link between *Polygonatum cyrtonema* polysaccharides and the mitigation of ulcerative colitis, through reducing oxidative stress and inflammation. An *in vivo* study demonstrated a synergistic therapeutic effect of applied extracts and intestinal microbiota, indicating them as promising candidates for UC therapy through microbiota-mediated mechanisms.

The editors believe that the research and review articles within this Research Topic outline the potential and current limitations of natural bioactive compounds from plants and mushrooms, facilitating the identification of existing knowledge gaps and future research opportunities.
